# From confusion to clarity: a case report of hypertensive and autoimmune encephalopathy in an elderly woman

**DOI:** 10.1097/MS9.0000000000002465

**Published:** 2024-08-14

**Authors:** Pratik Adhikari

**Affiliations:** B.P. Koirala Institute of Health Sciences, Dharan, Nepal

**Keywords:** autoimmune encephalitis, case report, elderly, hypertensive encephalopathy, immunotherapy

## Abstract

**Introduction and importance:**

Hypertensive encephalopathy is a critical condition characterized by acute hypertension-induced cerebral dysfunction, while autoimmune encephalitis involves immune-mediated neuronal damage. Distinguishing between these entities is crucial due to overlapping clinical features and distinct management approaches.

**Case presentation:**

The authors present a case of a 70-year-old woman with poorly controlled hypertension who initially presented with confusion and severe headache. Despite treatment for a hypertensive emergency, including intravenous labetalol, her neurological status deteriorated. She developed seizures and fever, prompting further investigations. Initial imaging and cerebrospinal fluid (CSF) analysis suggested hypertensive encephalopathy, but negative microbiological findings and persistent symptoms necessitated consideration of autoimmune causes.

**Clinical discussion:**

Clinical evaluation, EEG findings, and autoimmune panels were pivotal in diagnosing autoimmune encephalitis, supported by positive anti-NMDA receptor antibodies. Prompt initiation of high-dose intravenous immunoglobulin (IVIG) led to clinical improvement, underscoring the role of targeted immunotherapy.

**Conclusion:**

This case highlights the diagnostic complexities and therapeutic challenges of hypertensive and autoimmune encephalopathy overlap in elderly patients. Early recognition and tailored immunotherapy were instrumental in achieving favorable outcomes, advocating for a multidisciplinary approach to managing such complex neurological conditions.

## Introduction

Highlights

- The case highlights the challenge of distinguishing hypertensive encephalopathy from autoimmune encephalitis due to overlapping features.
- Initial treatment for hypertensive crisis expanded to include broad-spectrum antibiotics and IVIG, given the worsening clinical status and suspected autoimmune involvement.
- Prompt immunotherapy, including IVIG, led to significant recovery despite a grim initial prognosis with mechanical ventilation and neurological decline.
- Multidisciplinary collaboration was crucial in diagnosing autoimmune encephalitis and optimizing the patient’s long-term outcomes.

Hypertensive encephalopathy represents a critical neurological emergency characterized by acute elevations in blood pressure leading to cerebral edema and dysfunction^1^. This condition typically manifests with symptoms such as severe headache, altered mental status, and seizures, necessitating prompt management to prevent irreversible brain damage^2^. While hypertension remains a primary risk factor, emerging evidence suggests that autoimmune encephalitis, a group of inflammatory disorders affecting the CNS, can present with overlapping clinical features, posing diagnostic challenges, particularly in elderly patients with multiple commodities^3,4^.

Autoimmune encephalitis encompasses a spectrum of disorders mediated by autoantibodies targeting neuronal cell surface or synaptic proteins, resulting in diverse neurological manifestations including cognitive decline, behavioral changes, and seizures^5^. Diagnosis requires a comprehensive approach integrating clinical evaluation, EEG findings, CSF analysis, and autoimmune panels to identify specific antibodies indicative of autoimmune etiologies^6,7^.

This case report explores the diagnostic and therapeutic dilemmas encountered in an elderly woman initially presenting with symptoms suggestive of hypertensive encephalopathy, ultimately diagnosed with autoimmune encephalitis, highlighting the importance of timely recognition and tailored immunotherapy in optimizing patient outcomes^8^.

I am writing by the SCARE checklist. By the SCARE 2023 guideline (Sohrabi *et al.*, 2023), the methodology for reporting surgical case details was strictly adhered to in this study ^9^.

## Case presentation

A 70-year-old woman with a past medical history of hypertension but noncompliant with medications presented to the emergency department with a 2-day history of progressive confusion and worsening headaches. Her family reported she had become increasingly disoriented and forgetful, struggling to recognize familiar faces and places.

Her confusion began gradually, becoming more pronounced each day, with her headaches intensifying in severity over the same period. Additionally, she developed a stiff neck and increasing lethargy, which persisted without any periods of relief. These symptoms were not exacerbated or relieved by any specific factors, and there was no history of fever, recent travel, or illicit drug use to provide further clues.

On initial presentation, the patient was in acute distress. Vital signs were significant for severe hypertension (blood pressure 220/140 mmHg) and a Glasgow Coma Scale (GCS) score of 7 (out of 15). Neurological examination revealed disorientation to time, place, and person. The patient’s obtunded state made assessing the motor and sensory functions challenging.

**Figure 1:**
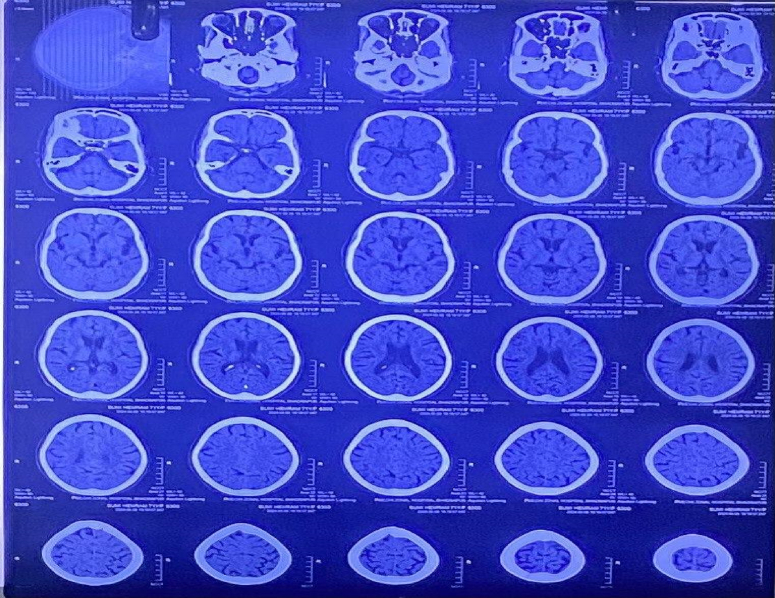
Head CT scan showing bilateral prominent cortical sulci, sylvian fissures, basal cisterns, and ventricles, indicative of cerebral atrophy.

Laboratory investigations revealed significant findings, with the complete blood count (CBC) showing marked leukocytosis and anemia (Table 1). Radiological investigations highlighted cerebral atrophy on the head CT scan (Fig. 1), while cerebrospinal fluid (CSF) analysis indicated elevated protein and lymphocytic pleocytosis, suggestive of inflammation (Table 2 and Table 3).

**Table 1:** Laboratory investigations.

Test	Result	Reference range	Significance
White blood cell count	25 600/mm³	4500–11 000/mm³	Leukocytosis
Hemoglobin	8.6 g/dl	12–18 g/dl	Anemia
Platelet count	212 000/mm³	150 000–450 000/mm³	Coagulopathy (borderline)
Sodium (Na)	144.5 mEq/l	135–145 mEq/l	Within normal limits
Potassium (K)	4.14 meq/l	3.5–5.5 meq/l	Within normal limits
Blood urea nitrogen (BUN)	40 mg/dl	15–45 mg/dl	Within normal limits
Creatinine (Cr)	0.77 mg/dl	0.4–1.4 mg/dl	Within normal limits
Liver function tests	Elevated		Suggestive of hepatic involvement

**Table 2:** Radiological investigations.

Test	Finding	Significance
Head CT Scan	Bilateral prominent cortical sulci, Sylvian fissures, basal cisterns, ventricles - suggestive of cerebral atrophy	May indicate underlying predisposition

**Table 3:** Cerebrospinal fluid (CSF) findings.

Test	Result	Significance	Helps rule out
Total leukocyte count (TLC)	10	Pleocytosis (mildly elevated white blood cells)	Viral meningitis (can be normal or mildly elevated), Bacterial meningitis (typically much higher WBC)
Differential leukocytes (DLC)	Neutrophils: 0%, Lymphocytes: 100%	Lymphocytic pleocytosis	Bacterial meningitis (typically neutrophilic pleocytosis)
CSF protein	425 mg/dl	Elevated protein	Viral meningitis (can be normal or mildly elevated)
CSF sugar	79 mg/dl	Within normal limits	Bacterial meningitis (typically low sugar)
CSF gram stain	Scanty pus cells, no bacteria seen	Negative for bacteria	Bacterial meningitis
CSF AFB stain	AFB not seen	Negative for tuberculosis	Tuberculous meningitis
CSF ADA	15 U/l	Within normal limits	Tuberculous meningitis (often elevated)

We made a differential diagnosis of hypertensive encephalopathy, central nervous system infection (e.g. viral or bacterial meningitis), autoimmune encephalitis, intracranial mass lesion, and toxic-metabolic encephalopathy. To evaluate these possibilities, we performed a head CT scan to assess for intracranial mass lesion, hemorrhage, or cerebral atrophy. A lumbar puncture and CSF analysis were conducted to evaluate for infection or inflammatory processes, and an EEG was performed to assess for encephalopathy or seizure activity.

### Clinical course and management

Given the severe hypertension, altered mental status, and neurological exam findings, hypertensive encephalopathy was suspected. Intravenous labetalol(20 mg bolus followed by continuous infusion at 1–2 mg/min) was initiated for blood pressure control. The patient was emergently admitted to the ICU due to her deteriorating mental status.

In the ICU, the patient experienced one episode of seizure, requiring anticonvulsant medication. Levetiracetam 1000 mg IV twice daily was administered. Her GCS score further declined to 3, and she developed a fever. Broad-spectrum antibiotics (ampicillin 2 g IV every 4 h), antiviral therapy with acyclovir 10 mg/kg IV every 8 h, corticosteroids (dexamethasone 10 mg IV every 6 h), and empiric antifungal therapy (fluconazole 800 mg IV daily) were initiated due to concern for meningoencephalitis. A lumbar puncture revealed elevated white blood cells and protein suggestive of inflammation but negative cultures for bacteria, viruses, and tuberculosis.

Despite aggressive management, the patient’s condition continued to deteriorate. On the second day of ICU admission, she became hemodynamically unstable with a significant drop in blood pressure (160/80 mmHg). Intubation and mechanical ventilation were initiated. On the third day of ICU admission, the patient’s condition remained critical, but an improvement in blood pressure control was noted. A neurology consult was requested, and an extended review of the patient’s history and clinical course was conducted. Given the resource-limited setting and the inability to perform an MRI, a diagnostic approach relying on clinical signs and available tests was essential.

Due to the persistence of symptoms and the critical need for a more precise diagnosis, additional diagnostic tools available in the setting were utilized. An electroencephalogram (EEG) was performed, which showed diffuse slow-wave activity suggestive of encephalopathy but without epileptiform discharges. This finding supported the diagnosis of hypertensive encephalopathy but did not rule out other potential underlying causes.

Given the evolving picture and fever, the team considered a broader differential diagnosis, including autoimmune encephalitis. A detailed autoimmune and paraneoplastic antibody panel was sent to an external laboratory, which provided results despite the delay. In the interim, the patient remained on mechanical ventilation, and anticonvulsant therapy was optimized with levetiracetam 1500 mg IV twice daily.

On the fourth day, empirical immunotherapy was initiated with high-dose intravenous immunoglobulin (IVIG) 0.4 g/kg/day for 5 days due to the high clinical suspicion of an autoimmune component. This decision was based on the patient’s lack of response to initial treatments and the need to address possible autoimmune encephalitis.

By the seventh day, the patient showed signs of clinical improvement. Her GCS score improved to 10, and she began to follow simple commands. The fever subsided, and repeat lumbar punctures showed decreased CSF white blood cells and protein levels, suggesting a response to the immunotherapy.

Over the following week, the patient continued to improve. She was gradually weaned off mechanical ventilation and transitioned to oral antihypertensives. Amlodipine 5 mg once daily and Lisinopril 10 mg once daily were administered. A multidisciplinary team closely monitored her progress, including neurology, infectious disease, and rheumatology. A week later, the autoimmune panel returned positive for anti-NMDA receptor antibodies, confirming the diagnosis of autoimmune encephalitis.

### Further management and follow-up

After 2 weeks, the patient was discharged from the ICU and transferred to a rehabilitation facility for continued physical and cognitive therapy. A tailored immunosuppressive regimen with oral corticosteroids (prednisone 1 mg/kg/day) and azathioprine 100 mg/day was initiated to prevent relapse of autoimmune encephalitis. Her blood pressure was controlled with a combination of ACE inhibitors (Lisinopril 10 mg once daily) and calcium channel blockers (Amlodipine 5 mg once daily), and she was educated extensively on the importance of medication adherence.

Six months postdischarge, the patient returned for a follow-up appointment. She exhibited significant cognitive recovery, with improved memory and orientation. Blood pressure was well-controlled, and no further episodes of confusion or seizure activity occurred. She continued her immunosuppressive therapy without significant side effects.

## Discussion

The presented case underscores the intricate interplay between hypertensive and autoimmune encephalopathy, reflecting the diagnostic challenges posed by overlapping clinical presentations. Hypertensive encephalopathy is characterized by sudden elevations in blood pressure leading to cerebral edema and subsequent neurological dysfunction, necessitating aggressive blood pressure control and close monitoring^1,2^. In elderly patients, the clinical picture can be further complicated by noncompliance with antihypertensive medications, as seen in our patient, exacerbating the risk of acute neurological complications^3^.

Autoimmune encephalitis, conversely, represents a relatively rare but increasingly recognized cause of encephalopathy, involving immune-mediated attacks on neuronal antigens. The clinical spectrum ranges from subacute cognitive decline to fulminant encephalopathy, often necessitating urgent immunomodulatory therapy for improved outcomes^4,5^. Differential diagnosis in suspected cases includes extensive laboratory testing for specific autoantibodies, such as anti-NMDA receptor antibodies, which are crucial in guiding targeted therapy and prognostication^6^.

In our case, initial diagnostic considerations centered around hypertensive encephalopathy given the patient’s history of poorly controlled hypertension and acute neurological decline. However, persistent symptoms despite optimal blood pressure management prompted further investigations, including EEG and CSF analysis, revealing characteristic findings suggestive of autoimmune etiology^7^. The delay in definitive diagnosis highlights the challenges posed by limited diagnostic resources and underscores the critical role of clinical suspicion and multidisciplinary collaboration in navigating complex neurological presentations^8,10^.

Treatment strategies in autoimmune encephalitis involve early initiation of immunotherapy, including corticosteroids, IVIG, and rituximab, aimed at suppressing autoimmune activity and promoting neurological recovery^11^. Our patient’s clinical improvement following high-dose IVIG underscores the efficacy of targeted immunotherapy, emphasizing the need for timely intervention in suspected autoimmune-mediated encephalopathies^12^.

In conclusion, this case highlights the diagnostic complexities and therapeutic challenges associated with overlapping presentations of hypertensive and autoimmune encephalopathy in elderly patients. It underscores the pivotal role of a multidisciplinary approach, advanced diagnostic modalities, and targeted immunotherapy in optimizing patient outcomes and preventing long-term neurological sequelae^13^.

### Limitations and key highlights

This case illustrates the complexity of managing a multifaceted neurological presentation, emphasizing the importance of considering autoimmune etiologies in hypertensive encephalopathy patients exhibiting atypical features such as persistent fever and elevated CSF white blood cells. Early recognition and intervention, supported by a multidisciplinary approach, were crucial in managing this patient’s condition. The initial limitations in diagnostic resources were overcome by utilizing tools such as EEG and extended laboratory testing, which ultimately guided appropriate immunotherapy. This case underscores the necessity of adapting clinical strategies to evolving patient conditions. It highlights the potential for recovery even in severe presentations of hypertensive and autoimmune encephalopathy with timely and aggressive treatment.

## Conclusion

This case underscores the intricate interplay between hypertension and autoimmune encephalopathy, presenting a diagnostic challenge compounded by the patient’s noncompliance with hypertension management. Despite aggressive treatment including blood pressure control, broad-spectrum antibiotics, and immunotherapy, the patient’s condition continued to deteriorate initially. The ultimate diagnosis of autoimmune encephalitis highlights the importance of considering autoimmune etiologies in patients with atypical neurological presentations. This case also emphasizes the critical role of a multidisciplinary approach and the adaptation of clinical strategies in managing complex neurological conditions in resource-limited settings.

## Ethical approval

Since the study is a case-report, we did not obtain ethical approval.

## Consent

Written informed consent was obtained from the patient for publication and any accompanying images. A copy of the written consent is available for review by the Editor-in-Chief of this journal on request.

## Source of funding

Not applicable.

## Author contribution

P.A.: provided us with data and materials from the archive and their notes, wrote the manuscript, collected the images and put them in perspective according to the timeline of the case, and reviewed the manuscript and did final editing. All the authors read the final manuscript and approved the case.

## Conflicts of interest disclosure

The authors declare that they have no financial conflict of interest with regard to the content of this report.

## Research registration unique identifying number (UIN)

This is a cross-sectional involving a human subject, so registration of the research study was done.

1. Registry used: Researchregistry.com.
2. Unique identifying number or registration ID: researchregistry10406.

## Guarantor

Pratik Adhikari is the guarantor of the study.

## Data availability statement

The datasets supporting the conclusions of this article are included within the article.

## Provenance and peer review

Not commissioned or externally peer-reviewed.
